# Seeing and sensing the heart: integrating non-coding RNA biomarkers with imaging in cardiovascular medicine

**DOI:** 10.1093/ehjimp/qyaf166

**Published:** 2026-01-09

**Authors:** Jan Alphard Kleeberger, Ludovica Di Venanzio, Natalia Atzemian, Frank Ruschitzka, Francesco Paneni

**Affiliations:** Department of Cardiology, University Heart Center; University Hospital Zurich, Zurich CH-8091, Switzerland; Center for Translational and Experimental Cardiology (CTEC), Department of Cardiology, University Hospital Zurich, University of Zurich, Wagistrasse 12, 8952 Schlieren, Zurich, Switzerland; Department of Cardiology, University Heart Center; University Hospital Zurich, Zurich CH-8091, Switzerland; Center for Translational and Experimental Cardiology (CTEC), Department of Cardiology, University Hospital Zurich, University of Zurich, Wagistrasse 12, 8952 Schlieren, Zurich, Switzerland; Center for Translational and Experimental Cardiology (CTEC), Department of Cardiology, University Hospital Zurich, University of Zurich, Wagistrasse 12, 8952 Schlieren, Zurich, Switzerland; Department of Cardiology, University Heart Center; University Hospital Zurich, Zurich CH-8091, Switzerland; Center for Translational and Experimental Cardiology (CTEC), Department of Cardiology, University Hospital Zurich, University of Zurich, Wagistrasse 12, 8952 Schlieren, Zurich, Switzerland; Department of Cardiology, University Heart Center; University Hospital Zurich, Zurich CH-8091, Switzerland; Center for Translational and Experimental Cardiology (CTEC), Department of Cardiology, University Hospital Zurich, University of Zurich, Wagistrasse 12, 8952 Schlieren, Zurich, Switzerland

**Keywords:** non-coding RNA, cardiovascular imaging, echocardiography, cardiac magnetic resonance, molecular biomarkers, heart failure

## Abstract

Non-coding RNA (ncRNA), including microRNA, long non-coding RNA, and circular RNA, are epigenetic signals acting as upstream regulators in several cardiovascular disease processes. This review explores how ncRNA profiling complements non-invasive cardiac imaging modalities by providing biological insights into structural and functional phenotypes. In conditions such as hypertensive heart disease and aortic stenosis, ncRNAs like microRNA (miR)-29 and miR-155 correlate with left ventricular hypertrophy and fibrosis. In dilated cardiomyopathy and heart failure, circulating miR-150–5p, miR-21, and LIPCAR associate with disease severity and prognosis beyond well-established echocardiographic prognosticators. Post-infarction remodelling has been linked to dynamic changes in miR-155, miR-143, and miR-150, while atrial disease and atrial fibrillation progression are reflected in distinct miRNA profiles. In valve disease, miR-206 levels mirror functional recovery after transcatheter aortic valve implantation, while they associate with right-ventricular dysfunction in the setting of pulmonary hypertension. Cardiac MRI studies have shown that ncRNA such as miR-29a and has-Chr8:96 may distinguish pathologies including hypertrophic cardiomyopathy and myocarditis. In nuclear imaging, circ-MBOAT2 and miR-495 correlate with myocardial perfusion in chronic total occlusion, and exosomal miRNA may support functional stratification. CT imaging may also benefit from ncRNA biomarkers such as miR-3154 in vascular disease. Despite promises, standardization and prospective validation remain crucial for clinical translation. Taken together, ncRNA enrich imaging phenotypes by illuminating molecular underpinnings, enhancing prognostication, and offering potential targets for personalized cardiovascular care.

## Graphical Abstract

**Graphical Abstract:**
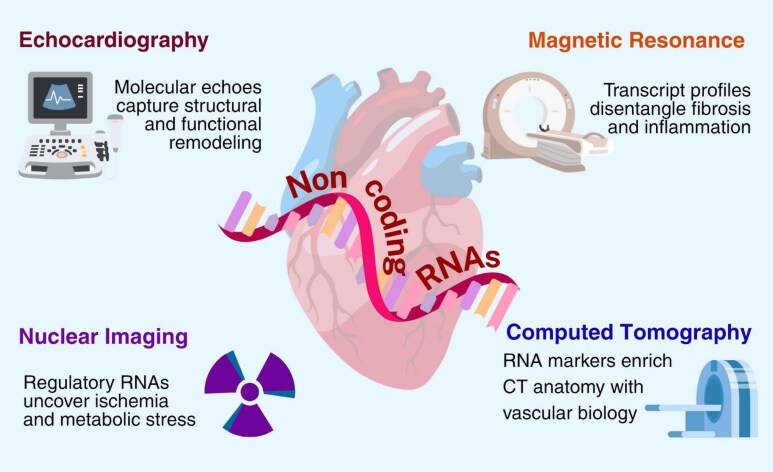

## Introduction

In recent decades, the rising global burden of cardiovascular diseases (CVDs) has underscored the critical need not only for novel therapeutic strategies but also for diagnostic approaches capable of achieving earlier and more precise disease detection.^1^ Traditional diagnostic tools, though valuable, often fail to detect subclinical stages or capture the complex, multi-factorial nature of CVD. Early and accurate detection is essential for improving patient outcomes, as it enables timely initiation of therapy, facilitates individualized risk stratification, and may ultimately reduce morbidity and mortality by preventing progression to advanced disease.

In modern cardiology, the reliance on invasive imaging modalities such as invasive coronary angiography (ICA), while highly informative, remains limited by patient discomfort, procedural risks, and cumulative radiation exposure. As a result, there has been a progressive shift towards non-invasive imaging techniques, which provide safer and often more patient friendly alternatives for the detection and monitoring of diseases such as ischaemic heart disease. Widely used approaches include transthoracic echocardiography (TTE), complemented by more advanced modalities such as single-photon emission computed tomography (SPECT) and cardiac magnetic resonance (CMR), which offer advanced phenotyping in cardiac remodelling, cardiomyopathies, and heart failure (HF).^2^ This transition reflects a broader paradigm in cardiovascular medicine, where precision diagnostics aim to balance accuracy with safety, while minimizing invasiveness and improving patient outcomes^3,4^

While advances in non-invasive imaging have significantly improved diagnostic precision and patient stratification in CVD, these modalities primarily capture structural and functional alterations that often occur downstream of molecular dysregulation. To achieve truly personalized medicine, complementary biomarkers are required to capture the upstream biological processes that drive disease onset and progression.

In this context, non-coding RNAs (ncRNAs) have emerged as critical regulators of cardiovascular pathophysiology and are increasingly recognized as a promising class of molecular biomarkers and therapeutic targets.^5^ Although only ∼2% of the genome encodes proteins, the vast majority consists of non-coding transcripts,^6^ highlighting their pervasive role in gene regulation. Advances in high-throughput sequencing, bioinformatics, and molecular biology have substantially deepened our understanding of ncRNAs biogenesis, function, and regulatory networks.^7,8^ At the same time, significant investment from both academia and industry has accelerated the translation of these discoveries into innovative therapeutic and diagnostic strategies. Collectively, these developments position ncRNAs as a concrete and rapidly evolving frontier in cardiovascular medicine.

ncRNAs are broadly classified into small ncRNAs, including microRNAs (miRNAs), and long ncRNAs (lncRNAs), which encompass both linear and circular species (circRNAs) with diverse structural and regulatory properties. Among these, miRNAs represent the most extensively studied subclass, owing to their well-established involvement in cardiac pathophysiology in conditions such as dilated cardiomyopathy (DCM) and obesity-related cardiomyopathy.^9,10^ Although miRNAs dominate current research, increasing evidence points to crucial roles for lncRNAs and circRNAs in atherosclerosis, cardiac hypertrophy, fibrosis, and ischaemic injury, thereby broadening the landscape of ncRNAs-mediated regulation in CVD.^11–14^ Notably, early phase clinical trials with miRNAs-based therapeutics, such as antimiR-92a in ischaemic heart disease and CDR132L in HF, highlight the feasibility and translational promise of RNA-targeted interventions.^5,10,15,16^

In this review, we explore how non-invasive imaging modalities can be integrated with ncRNAs-based molecular profiling to provide a more comprehensive understanding of CVD. By combining structural and functional assessment with insights into upstream molecular regulation, such multi-modal strategies may enhance diagnostic precision, refine risk stratification, and support the development of truly personalized approaches in cardiology.

### ncRNAs and echocardiography

Echocardiography is the clinical imaging backbone for the assessment of cardiac structure and function, from ejection fraction (EF) and chamber dimensions to myocardial deformation and diastolic indices.^17^ In parallel, ncRNAs have emerged as regulators of hypertrophy, fibrosis, apoptosis, and inflammation, processes that echocardiography routinely tracks at the macroscopic organ level. This convergence creates an opportunity: circulating or tissue ncRNA signatures may complement echocardiographic phenotypes by capturing upstream biology that precedes changes in left ventricular ejection fraction (LVEF), global longitudinal strain, diastolic function parameters, left atrial (LA) strain, and right-ventricular function.

Across a range of cardiovascular conditions, circulating and ncRNAs enriched in the myocard—primary miRNAs, with growing evidence for lncRNAs and circRNAs—have been associated with echocardiographic markers of hypertrophy, fibrosis, systolic dysfunction, atrial remodelling, and right-sided load. The sections that follow synthesize these data thematically, highlight methodological caveats, and consider translational prospects.

#### Hypertrophy and fibrosis

Left ventricular (LV) remodelling, driven by chronic pressure overload such as arterial hypertension or aortic stenosis (AS), involves profound structural and molecular adaptations. Among these, ncRNAs have emerged as key regulators orchestrating hypertrophy and fibrosis. The miR-29 family has been identified for its anti-fibrotic function. Circulating levels of miR-29 have been shown to correlate with LV hypertrophy in hypertensive patients, aligning with echo-derived increases in LV mass index.^18^ Similarly, the same group reported that miR-155 levels correlated with blood pressure and indices of target organ damage, supporting its role as a mediator of hypertrophic remodelling.^19^

Circulating levels of the miR-29 family, known for their anti-fibrotic regulatory role, have been shown to correlate with LV hypertrophy in hypertensive patients, mirroring echocardiographic increases in LV mass index.^18^ Similarly, miR-155 levels correlate with blood pressure and indices of target organ damage, supporting a role in hypertrophic remodelling.^19^

In AS, circulating miR-19b has been associated with myocardial collagen cross-linking and HF, linking this molecular signal to concentric hypertrophy and myocardial stiffness detected by echocardiography.^20^ Moreover, distinct patterns of circulating miRNAs have been identified among different AS subgroups, revealing biological heterogeneity that is not apparent from echocardiographic gradients alone.^21^ Myocardial miRNA signatures may also influence long-term ventricular remodelling after valve intervention, suggesting potential value for predicting LV mass regression and diastolic recovery.^22^

In hypertrophic obstructive cardiomyopathy, serum circular RNAs (circRNAs) have emerged as promising biomarkers, effectively distinguishing affected patients from those with non-obstructive hypertrophic cardiomyopathy and from healthy controls.^23^ In addition to circRNAs, have been associated with increased septal thickness, greater obstruction severity, and impaired myocardial strain, supporting its role as an indicator of both hypertrophy and fibrosis.^24^

Overall, these ncRNAs offer a molecular lens through which the structural and functional changes captured by echocardiography can be interpreted, strengthening the link between circulating biomarkers and the pathobiology of pressure-overload remodelling.^18–24^

#### HF and DCM

DCM and systolic HF present with LV dilation, impaired myocardial strain, and reduced EF. A diagnostic model combining three miRNAs (miR-130b-3p, miR-150–5p, and miR-210–3p) with clinical variables accurately identified patients with idiopathic DCM and severely reduced EF. Among 179 initially screened miRNAs, 14 were associated with echocardiographic features, and three miRNAs mentioned before showed strong predictive value when integrated into a diagnostic model. These findings suggest a novel biomarker tool with high potential for improving risk stratification in idiopathic DCM.^25^ In another multi-centre case-control study, specific circulating circRNAs were differentially expressed across aetiologies of DCM, including Lamin A-related and ischaemic subtypes. Notably, four circRNAs (hsa_circ_0003258, hsa_circ_0051238, hsa_circ_0051239, and hsa_circ_0089762) showed significant overexpression and correlated with systolic and diastolic echocardiographic parameters. This underscores their potential to differentiate phenotypes that cannot be fully distinguished using clinical assessment or echocardiography alone.^26^ Several ncRNAs also provide prognostic information. For instance, miR-150–5p was identified and validated as a marker of advanced HF in a prospective genome-wide study.^27^ Building on prior evidence, the lncRNA LIPCAR has been shown to predict outcomes independently of kidney function, underscoring its potential to complement serial echocardiographic evaluation of cardiac remodelling.^28^ Elevated circulating levels of miR-143 and miR-145 have been associated with LV systolic dysfunction, reflecting structural remodelling detectable by echocardiography.^29^ Likewise, miR-21 correlates with inflammatory markers in chronic HF and may reflect diastolic dysfunction, which is often assessed through echocardiographic parameters such as *E*/*e*′ and LA enlargement.^30^ These findings support the integration of miRNA profiling with echocardiographic assessment to capture both functional and molecular dimensions of cardiac remodelling. Hence, ncRNA profiles may refine risk stratification in DCM and HF by providing information on underlying biology and prognosis that complements echo-derived measures such as EF and GLS.

#### Ischaemic injury and post-myocardial infarction remodelling

Maladaptive remodelling is a key determinant of outcomes in post-myocardial infarction (MI). The persistence of inflammatory signalling reflected at the molecular level has been associated with unfavourable structural changes. In particular, the inability to suppress miR-155 expression after ST-elevation MI has been linked to adverse remodelling, characterized by increases in LV end-diastolic and end-systolic volumes as observed on follow-up echocardiography.^31^ Similarly, elevated levels of miR-150 in the early post-infarction phase have been shown to predict the transition to HF, suggesting its involvement in the maladaptive cascade that leads to progressive systolic dysfunction.^32^ Conversely, dynamic regulation of miR-143 appears to capture favourable myocardial adaptation, as acute elevations in this miRNA during the subacute phase correlate with improvements in LVEF, indicating its potential role in identifying patients undergoing reverse remodelling.^33^ Taken together, these findings suggest that early post-MI profiling of circulating miRNAs may help distinguish patients at risk for progressive LV dilation and dysfunction from those more likely to experience structural and functional recovery, as assessed by serial echocardiographic imaging.

#### Atrial remodelling and atrial fibrillation

NcRNAs have also been implicated in atrial disease. In endurance athletes, acute atrial remodelling following strenuous exercise, such as marathon running, has been shown to induce transient shifts in circulating miRNA expression, suggesting that miRNA levels may reflect dynamic changes in atrial structure and wall stress.^34^ Population-based data from the Framingham Offspring Study further support this concept, with specific plasma miRNAs found to associate with echocardiographic markers of atrial size and function, as well as prevalent atrial fibrillation.^35^ These associations have been strengthened by studies incorporating both intra-cardiac and peripheral sampling, which identified miR-20b-5p and miR-330–3p as biomarkers of LA enlargement and disease progression in patients with atrial fibrillation.^36^ The ncRNA mirror atrial remodelling phenotypes on echocardiography and may help identify individuals at higher risk of atrial fibrillation progression.

#### Valve interventions and transcatheter aortic valve implantation

Predicting functional recovery following transcatheter aortic valve implantation (TAVI) remains clinically challenging, particularly in patients with borderline or reduced baseline LVEF. Recent findings have highlighted the potential of circulating ncRNAs to serve as early, non-invasive biomarkers for myocardial recovery. Among these, miR-206 has emerged as a promising candidate: elevated levels of this muscle-specific miRNA prior to or shortly after TAVI were associated with lower LVEF, while patients exhibiting a post-procedural decline in miR-206 showed greater improvements in ventricular function on serial echocardiography.^37^ These observations suggest that down-regulation of miR-206 may reflect favourable reverse remodelling and may parallel structural and functional improvement captured by imaging. Additional evidence has reinforced this paradigm. The miR-122–5p, a miRNA involved in metabolic and apoptotic signalling, was recently associated with a lack of LVEF improvement after TAVI and shown to regulate cardiomyocyte viability via extracellular vesicle–mediated mechanisms.^38^ In this context, persistently high levels of miR-122–5p may indicate ongoing myocardial injury or suboptimal remodelling. Furthermore, miR-223, which plays roles in inflammation and platelet function, has also been proposed as a prognostic marker, with its expression linked to adverse outcomes after TAVI and potential utility in early risk stratification.^39^ Collectively, these studies support the use of ncRNA profiling as a complementary tool to echocardiography, offering molecular insights into post-TAVI myocardial adaptation and facilitating more individualized post-procedural surveillance strategies.

#### Right-heart load and pulmonary hypertension

In patients with left heart disease complicated by pulmonary hypertension (PH), ncRNAs are increasingly recognized as molecular correlates of right ventricular (RV) pressure overload and dysfunction. Circulating miR-206 has been shown to reflect PH severity, correlating with echocardiographic measures such as tricuspid regurgitant velocity and tricuspid annular plane systolic excursion, both of which are routinely used to assess RV function and pulmonary pressure in clinical practice.^40^ These findings suggest that miR-206 may serve as a mechanistic link between myocardial stress and right-sided remodelling. Further evidence for the utility of ncRNAs in PH stratification comes from a recent multi-cohort study, which identified distinct diagnostic miRNA signatures capable of differentiating between PH subtypes, including group 2 PH secondary to left heart disease and pre-capillary forms such as pulmonary arterial hypertension.^41^ In that study, miRNA profiles not only supported hemodynamic classification but also aligned with functional and structural right-heart parameters on imaging, highlighting their potential to augment conventional echocardiographic evaluation. Together, these data underscore the emerging role of circulating miRNAs as adjunctive biomarkers in the phenotyping and monitoring of PH, particularly in complex cases where RV function and pulmonary pressures must be interpreted in the context of underlying left-sided pathology.

#### Acute global dysfunction and post-resuscitation

In the setting of cardiac arrest, acute alterations in circulating ncRNAs have been shown to mirror the severity of myocardial dysfunction. Elevated levels of miR-21 were found to correlate with LV systolic impairment on echocardiography in patients who had been successfully resuscitated, suggesting a link between molecular stress responses and post-arrest myocardial stunning.^42^ These findings indicate that early miRNA profiling may capture transient, potentially reversible myocardial dysfunction, offering prognostic value beyond imaging alone. In particular, dynamic changes in miR-21 may serve as adjunctive biomarkers for identifying patients at risk of poor cardiac recovery following resuscitation.

In summary, integrating ncRNAs with echocardiographic phenotyping offers a promising avenue to capture biological processes that imaging alone cannot fully resolve. Across various cardiovascular conditions, ncRNAs consistently mirror structural and functional remodelling and may provide earlier or more specific signals than conventional parameters. Their ability to reflect cross-ventricular or systemic involvement further underscores their potential clinical value. However, heterogeneity in study designs, small cohorts, and limited mechanistic validation remain important challenges. As emerging lncRNAs and circRNAs refine phenotypes beyond current imaging resolution, carefully designed studies will be essential to determine how these biomarkers can meaningfully enhance risk stratification and guide patient management alongside echocardiography.

### ncRNA and cardiac MRI

CMR has evolved into a cornerstone of modern cardiac imaging, combining precise quantification of ventricular volumes and function with detailed tissue characterization. Its capacity to detect inflammation, fibrosis, oedema, and perfusion defects has made it indispensable across diverse cardiovascular conditions.^43^ In cardiac amyloidosis, CMR offers unparalleled diagnostic accuracy, representing the most reliable non-invasive method for confirming the disease.^44^ Likewise, in ischaemic heart disease, assessment of peri-infarct ischaemia by CMR has been identified as a strong independent predictor of mortality and adverse cardiovascular outcomes, emphasizing its prognostic relevance.^45,46^

However, the clinical utility of CMR is still constrained by its limited availability in many centres. In this context, the integration of advanced cardiac imaging with molecular tools, particularly ncRNAs, offers a promising avenue for developing cost-effective, accessible, and disease-specific biomarkers. Such an approach could bridge the gap between molecular pathophysiology and structural imaging, enabling more precise diagnosis, refined prognostic assessment, and improved risk stratification in the era of precision medicine.

Evidence across multiple cardiovascular conditions supports this synergy. In patients with hypertrophic cardiomyopathy, circulating miRNA profiles, such as elevated miR-29a, have been linked to fibrosis-related structural remodelling, as reflected by myocardial wall thickness and LV mass index on both TTE and CMR imaging.^47^ In genetic cardiomyopathies such as muscular dystrophies, sex-specific differences in circulating miRNA levels have been observed, and these may serve as early functional markers that prompt targeted CMR for comprehensive structural and tissue characterization.^48^ This integrated paradigm also holds potential in inflammatory heart disease. Myocarditis, despite CMR's central role, remains challenging to diagnose owing to restricted imaging access and the risks of endomyocardial biopsy.^49^ Here, ncRNAs may offer a valuable adjunct: the ncRNA has-Chr8:96 has demonstrated excellent discriminatory capacity between myocarditis and MI (AUC 0.927), with high specificity maintained after adjustment for demographic, functional, and biochemical variables.^50^ This discovery originated in experimental autoimmune and viral myocarditis models in mice, where mmu-miR-721, a miRNA secreted by pathogenic Th17 cells, was selectively elevated in the plasma of animals with myocarditis but not in those with MI. A human homologue of this transcript, hsa-Chr8:96, was subsequently validated across four independent human cohorts of patients with biopsy- or CMR-confirmed myocarditis. In cardiac remodelling after acute MI, serial miRNA profiling has further revealed their value as dynamic biomarkers. Distinct expression patterns between patients with and without adverse LV remodelling, and variation between early (2-week) and later (6-week) post-acute MI phases, underscore their potential to monitor disease progression and recovery beyond the capabilities of imaging alone (*Supplementary data online, Ref. S51*).

### ncRNA and nuclear imaging (PET/SPECT)

Nuclear imaging modalities, such as positron emission tomography (PET) and SPECT, offer powerful insights into myocardial metabolism, perfusion, and inflammation. Recent studies have begun to bridge the gap between molecular signatures and functional imaging by investigating the role of ncRNAs in modulating or reflecting pathophysiological processes detectable via nuclear techniques, e. g. in terms of myocardial perfusion (*Supplementary data online, Ref. S52*). In patients with chronic total occlusion and stable coronary artery disease, circulating circRNAs and miRNAs were profiled by sequencing, and key candidates were evaluated for their relationship to myocardial perfusion assessed by SPECT (*Supplementary data online, Ref. S52*). They discovered a link between the improvement in myocardial perfusion after revascularization of chronic total occlusion lesions and circ-MBOAT2 and miR-495, hence identify a potential druggable target as well as a potential prognostic factor in this cohort. Another non-randomized, prospective study in patients with chronic coronary syndrome tested whether the use of various circulating miRNA might augment the diagnostic accuracy of myocardial perfusion imaging. This yielded a modest performance of miRNA biomarkers in discriminating between patients with and without myocardial ischaemia as determined by nuclear stress imaging techniques (*Supplementary data online, Ref. S53*). Using a comparable design, and measured miRNAs previously implicated in myocardial injury in patients undergoing myocardial SPECT. miR-21, miR-208a, and miR-499 concentrations determined at different time points around myocardial SPECT did not provide additional diagnostic benefit in differentiating patients in terms of functionally relevant chronic coronary syndrome (*Supplementary data online, Ref. S54*). Leveraging next-generation sequencing of miRNA enriched in circulating exosomes in patients with significant chronic coronary syndrome undergoing myocardial SPECT, a recent prospective non-randomized study from China unraveled miRNA-432–5p and miRNA-382–3p to be up-regulated in patients with abnormal myocardial perfusion (*Supplementary data online, Ref. S55*).

Nuclear imaging techniques in general are also useful in detecting metabolic and inflammatory activity in the body. Interestingly, miRNA sequencing indicated a regulation of miRNA associated with inflammation. This hints to an activation of inflammatory pathways in patients with desmoplakin and titin cardiomyopathy, which could not be detected in FDG PET-CT (*Supplementary data online, Ref. S56*).

Overall, some miRNAs were implicated with the findings of nuclear imaging techniques, particularly in relation to myocardial perfusion. This suggests their potential as future biomarkers, although validation studies are still lacking. Since nuclear imaging modalities also offer powerful insights into cardiac metabolism and inflammation, it may be worthwhile investigating ncRNAs that reflect pathophysiological processes detectable via nuclear techniques in these areas. To the best of our knowledge, no trials explored this connection to date. Most available studies are small, with heterogeneous radiotracers and endpoints, precluding reproducibility. Technical variation in quantification and tracer uptake also complicates standardization. Future research should focus on prospective protocols that align serial ncRNA sampling with SPECT or PET imaging to explore temporal relationships and diagnostic complementarity in inflammatory cardiomyopathies.

### ncRNAs and CT imaging

Computed tomography (CT) remains the primary non-invasive diagnostic modality for evaluating patients with suspected coronary artery disease and represents one of the most rapidly advancing areas in cardiovascular imaging. Its marked inter-individual variability has also made it a prime example of how artificial intelligence (AI) is transforming cardiology and, more broadly, clinical medicine enhancing diagnostic confidence, improve disease characterization, and facilitate more tailored medical therapy and patient management^46^ (*Supplementary data online, Refs. S57 and S58*).

Parallel to these developments, molecular biomarkers, particularly ncRNAs, are gaining recognition as complementary tools that add a biological dimension to imaging-based assessments. Recent integrated imaging studies support this approach, showing that complementing CT with detailed molecular analyses uncovers regulatory networks, such as miRNA-mediated pathways, that shape the calcific phenotype beyond what structural imaging can capture (*Supplementary data online, Ref. S59*). Consistent with this concept, recent clinical work has identified circulating miRNAs such as miR-134, miR-3135b, and miR-2861 as correlates of CT-derived coronary calcium burden and as discriminators of obstructive coronary disease in symptomatic patients (*Supplementary data online, Ref. S60*). Evidence from both cell-based and mouse models indicate that miR-222-3p enriched nanovesicles protect against ischaemia-reperfusion injury by stabilizing mitochondrial function and reducing oxidative stress. This protective response is driven by the miR-222-3p dependent regulation of cyp1a1 (*Supplementary data online, Ref. S61*). In abdominal aortic aneurysm, for instance, miR-3154 has been implicated as a pathogenetic factor with both therapeutic and prognostic relevance. This example illustrates how integrating CT with molecular profiling can link circulating RNA signatures to disease phenotype, thereby enhancing risk stratification and supporting precision medicine in CVD (*Supplementary data online, Ref. S62*). To the best of our knowledge, no studies correlated coronary CT data with the expression profiles of ncRNAs. Hence, studies examining this connection might help linking the phenotype with imaging findings, as illustrated by the study connecting miR-3154 with abdominal aortic aneurysm. Despite rapid technological progress, the integration of ncRNAs with CT-based phenotyping remains limited. This is partly because CT has traditionally been used to quantify structural features such as luminal stenosis, plaque burden, or calcification, whereas ncRNA studies often target dynamic biological processes that are not directly captured by CT. Moreover, the lack of longitudinal CT datasets linked to biospecimen repositories has restricted the field. As combined imaging–omics efforts expand, more robust evidence is expected, but current studies remain exploratory and involve relatively small cohorts.

#### Cardio-oncology and ncRNAs

Although not a primary focus of this review, there is growing interest in the role of ncRNAs in cancer therapy-related cardiac dysfunction. Pre-clinical *in vivo* models of anthracycline-induced cardiotoxicity have identified up-regulation of miR-34a in association with myocardial fibrosis and systolic impairment (*Supplementary data online, Ref. S63*). While human data are still limited, such miRNAs may serve as early indicators of cardiotoxicity and could complement echocardiographic monitoring in cardio-oncology clinics. Future integration of ncRNA profiling into surveillance algorithms may help detect early subclinical changes and guide cardioprotective strategies.

### Mechanistic insights

The intersection between ncRNAs and cardiac imaging is underpinned by the role of these molecules as upstream regulators of key pathophysiological pathways, namely fibrosis, hypertrophy, apoptosis, inflammation, and vascular remodelling. Mechanistically, the miR-29 family has been shown to directly target collagen genes (e.g. COL1A1, COL3A1), thereby suppressing fibrotic signalling cascades. These anti-fibrotic properties mirror late gadolinium enhancement patterns observed on CMR and correlate with increased LV mass index in echocardiographic assessments of hypertensive or AS-induced hypertrophy^18^ (*Supplementary data online, Ref. S64*). miR-155, a well-characterized immunomodulatory miRNA, contributes to maladaptive remodelling by sustaining macrophage activation and promoting cytokine-mediated damage following MI and in hypertensive heart disease. Lack of miR-155 down-regulation after MI correlates with progressive LV dilation and adverse remodelling on follow-up echocardiography.^31^ Tissue-specific expression of ncRNAs further supports their mechanistic relevance. Myocardial biopsy data have demonstrated that miRNA signatures not only correlate with imaging markers of recovery following TAVI but may actively govern the reverse remodelling process. For instance, miR-206 down-regulation post-TAVI has been linked to enhanced LVEF recovery, suggesting a causal role in myocardial plasticity.^37^ Similarly, in myocarditis, has-Chr8:96 has been identified as a specific molecular discriminator from MI, showing high diagnostic specificity and aligning with CMR findings in inflammatory heart disease.^50^ Temporal dynamics of ncRNAs expression also underscore their potential as early biomarkers. In patients undergoing TAVI or post-MI remodelling, longitudinal profiling has revealed that shifts in circulating miRNA levels, i.e. miR-150, miR-143, and miR-122-5p precede structural changes on imaging and may even anticipate symptom onset.^32,33,38^ These dynamic patterns reflect regulatory involvement in myocardial cell viability, inflammatory resolution, and extracellular matrix remodelling, processes which collectively shape imaging phenotypes observed weeks to months later. Collectively, these findings suggest that ncRNAs are not merely epiphenomena but active participants in cardiovascular remodelling. Their expression patterns provide mechanistic insights into the biological underpinnings of imaging-derived phenotypes, offering an additional molecular layer for interpreting structural and functional changes captured by echocardiography, CMR, and advanced imaging modalities.

### Clinical relevance and translational potential

**Figure 1:**
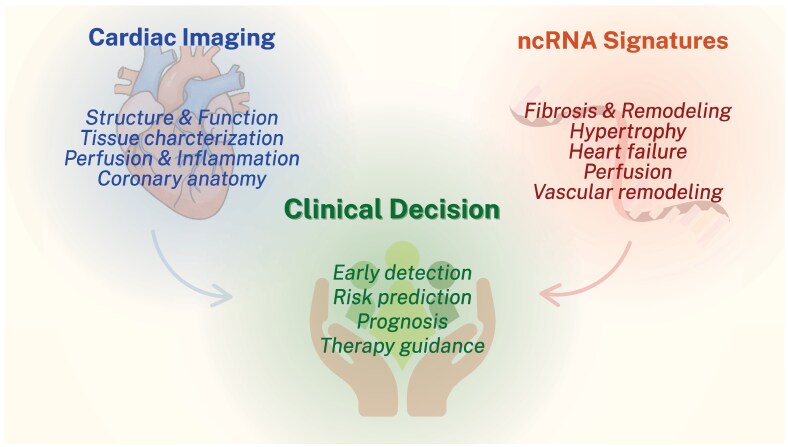
Complementary roles of cardiac imaging and non-coding RNA signatures in clinical decision-making. This schematic highlights the synergistic integration of cardiac imaging and ncRNA profiling. Imaging provides structural, functional, and anatomical insights, while ncRNA signatures reflect upstream molecular processes such as fibrosis, hypertrophy, and vascular remodelling. Together, these modalities support early detection, risk prediction, prognostic assessment, and individualized therapy guidance in CVD.

ncRNA as biomarkers hold significant promise for redefining clinical workflows in cardiovascular imaging by facilitating earlier detection, enhancing phenotypic precision, and improving risk stratification. Their intrinsic properties such as non-invasiveness and relative stability in the circulation make them particularly suitable for routine clinical use, especially in scenarios where imaging results are equivocal, or access is limited. *Figure 1* depicts the integration of cardiac imaging and ncRNA profiling. Beyond diagnostic augmentation, ncRNAs may also enable molecular discrimination between phenotypically similar entities, such as hypertrophic obstructive cardiomyopathy vs. hypertensive LV hypertrophy, or fibrotic vs. functional remodelling following MI. In more advanced imaging contexts, such as CMR or PET, ncRNAs may serve as gatekeepers for resource allocation, identifying patients most likely to benefit from comprehensive structural or tissue-level characterization. Furthermore, the potential of specific ncRNAs as therapeutic targets reinforces their translational relevance. Molecules such as miR-495, implicated in angiogenic signalling within chronic coronary syndromes, exemplify how biomarker discovery may inform both diagnosis and intervention. However, the clinical implementation of ncRNA-based tools remains constrained by methodological challenges. Standardization of pre-analytic protocols, assay harmonization, and cross-population validation are pre-requisites for widespread adoption. Despite the expanding body of evidence, the field is limited by small, observational studies that often lack mechanistic interrogation or prospective design. Redundancy in ncRNA-target interactions and overlapping expression profiles may further complicate interpretation and clinical decision-making. Crucially, there remains a paucity of large-scale, outcome-driven studies assessing whether the addition of ncRNA profiling substantively improves patient outcomes beyond those achieved with imaging alone. Addressing these gaps will be essential to realizing the full potential of ncRNA in cardiovascular diagnostics and therapy.

To guide integration into clinical workflows, we propose a conceptual ncRNA-imaging interface. In pre-imaging triage, plasma ncRNA signatures could help prioritize patients for advanced modalities. For example, by identifying those with suspected myocarditis or subclinical hypertrophy who may benefit from CMR. Post-intervention, such as after TAVI, serial measurement of miR-206 or miR-122-5p may signal myocardial recovery or stagnation, prompting tailored follow-up. Similarly, in HF clinics, combining miRNA panels with echocardiographic parameters could refine risk stratification and assist with therapy adjustment. This layered diagnostic strategy aligns with precision cardiology by integrating molecular and structural information into decision-making.

### Evidence limitations

While the current body of literature shows promising correlations between ncRNAs and cardiac imaging phenotypes, several limitations persist. Many studies are single-centre, exploratory cohorts with modest sample sizes, limited longitudinal data, and high analytical heterogeneity. The lack of assay standardization, divergent normalization strategies, and differences in sampling time points reduce reproducibility and hinder cross-comparison. Furthermore, only a subset of studies has incorporated multi-variate adjustment or assessed additive prognostic value beyond imaging. To ensure translational utility, robust multi-centre validation with harmonized protocols is urgently needed.

### Clinical integration pathway and future directions

To advance ncRNAs towards clinical implementation, a stepwise translational framework is required. This includes: (i) technical harmonization of ncRNA assays and reporting standards; (ii) multi-centre validation with diverse patient populations; (iii) integration into imaging-rich prospective studies to evaluate additive predictive value; and (iv) cost-effectiveness and feasibility analysis in real-world settings, ideally augmented by machine learning-based predictive models. Establishing centralized biobanks and multi-centre consortia with harmonized sampling, imaging, and outcome protocols will be critical to achieving reproducibility. As molecular imaging and precision medicine evolve, the combined use of circulating ncRNAs and cardiac imaging offers a promising path towards earlier diagnosis, individualized surveillance, and improved outcomes across a spectrum of CVD. Ultimately, the identification of functionally active ncRNAs may not only serve diagnostic purposes but also unlock novel therapeutic targets (*Table 1*).

**Table 1:** Echocardiographic findings and clinical insights of ncRNAs across cardiac pathological conditions.

Echocardiography				
Condition	ncRNA	Biological function	Echocardiographic findings	Clinical insights
Hypertrophy and fibrosis	miR-29, miR-155	Regulate fibrosis and hypertrophy	↑LV mass index	Reflect hypertrophy in hypertensive patients^18,19^
	miR19b	Modulates collagen cross-linking via LOX	↑ LV stiffness	Linked to aortic stenosis and HF^20,21^
	miR-4709-3p	Regulates myocardial remodelling	↓ LV mass post-AVR	Predicts hypertrophy regression^22^
	circTEMES6, circDNACJ6	circRNA biomarkers of hypertrophy	↑ IVST, LV mass index, and ↓ LVEF	Distinguish HOCM from non-obstructive HCM and controls^23^
	miR-221	Promotes fibrosis and hypertrophy	↑ Maximum interventricular septal thickness	Marker of HOCM severity^24^
			↑ Left ventricular mass index	
			↓ LVEF	
Heart failure and dilated cardiomyopathy	miR-130b-3p, miR-150-5p, miR-210-3p	Regulate remodelling pathways	↓ LVEF	Diagnostic panel for idiopathic DCM, predicts severe systolic dysfunction^25^
	hsa_circ_0003258 and hsa_circ_0051239	circRNAs reflecting DCM Aetiology (Lamin A-related, ischaemic)	↓ Early diastolic mitral annular velocity	Discriminate DCM subtypes beyond imaging findings^26^
	hsa_circ_0051238		↑ Septal atrial systolic mitral annular velocity	
	hsa_circ_0051238, hsa_circ_0051239		↓ LV outflow tract velocity	
	lncRNA LIPCAR	Regulates mitochondrial metabolism and remodelling	-	Prognostic marker in HF independent of renal function; increased after post-MI recovery and chronic HF
	miR143 and miR145	Reflect remodelling and cytoskeletal signalling	↓ LVEF	Associated with LV systolic dysfunction^29^
			↑ LV end-systolic dimension	
		Regulates inflammatory and stress-response pathways	↓ LVEF, RVEF, SV, and CI	Links inflammation and ventricular dysfunction in chronic HF and post-resuscitation settings; biomarker of myocardial injury^30,42^
			↑ LVPW, LVMI, and LVRI	
Ischaemic injury and post-MI remodelling	miR-155	Sustained inflammatory activation	↑ LVEDV and LVESV	Predicts adverse remodelling and HF progression post-MI^31^
	miR-150	Regulates remodelling and transition to HF	↓ EF recovery	Early post-MI predictor of maladaptive remodelling^32^
	miR-143	Reflects reverse remodelling	↑ LV functional recovery in the chronic phase	Biomarker predicting LV functional recovery in chronic phase after AMI^33^
Atrial remodelling, valve interventions, and PH	miR-1, miR-30a, and miR-133a	Reflect atrial stretch and electrical remodelling	↑ LA diameter	Associated with AF onset and progression^34^
	miR-106b, miR-26a-5p, miR-484, and miR-20a-5p	Regulate cardiac hypertrophy, inflammation, and fibrosis	Associated with LAFI	Linked to atrial remodelling and both prevalent and incident AF in population-based cohort^35^
	miR-20b-5p and miR-330-3p	Cardiac-specific regulation of atrial fibrosis	↑ LA diameter	Decreased levels associated with AF recurrence and remodelling progression after catheter ablation^36^
	miR-206	Cardiac-enriched miRNA regulating contractile remodelling	↓ LVEF, ↑ PASP, and ↑ LAD	Higher levels predict poorer LV recovery; post-TAVI reduction reflects improved function^37^; lower levels associate with higher pulmonary pressure; predicts PH with BNP and LAD^40^
	miR-122-5p	Endothelial EV miRNA regulating apoptosis	↓ LVEF improvement at 7 days and 6 months post-TAVI	Higher circulating levels associate with impaired LV recovery; proposed biomarker of persistent myocardial dysfunction after valve replacement^38^

LOX, lysyl oxidase; HOCM, hypertrophic obstructive cardiomyopathy; HCM, hypertrophic cardiomyopathy; AMI, acute myocardial infarction; AF, atrial fibrillation; EV, extracellular vesicles; AVR, aortic valve replacement; IVST, interventricular septal thickness; SV, stroke volume; CI, cardiac index; LVPW, left ventricular posterior wall thickness; LVMI, left ventricular mass index; LVRI, left ventricular remodelling index; LVEDV, left ventricular end-diastolic volume; LVESV, left ventricular end-systolic volume; LAFI, left atrial function index; PASP, pulmonary artery systolic pressure; LAD, left atrial diameter.

## Conclusion

This review highlights how ncRNAs complement established cardiovascular imaging modalities by adding a molecular layer that enhances phenotype interpretation. Through their involvement in processes such as fibrosis, hypertrophy, and inflammation, ncRNAs can contextualize structural and functional imaging findings and may augment diagnostic and prognostic assessment. However, their translation into imaging-guided clinical practice will require robust prospective studies, standardized analytical workflows, and clearer insight into their biological origins and temporal behaviour. Future work integrating ncRNA profiling with multi-modal imaging and clinical phenotypes will be essential to advance personalized cardiovascular care, with ncRNAs serving as molecular adjuncts rather than replacements for imaging.

## Lead author biography


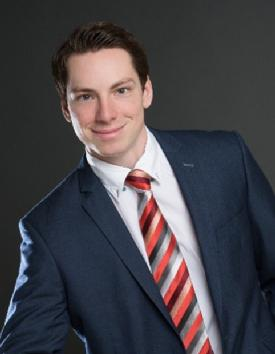


Jan Alphard Kleeberger, MD, MHBA, is an attending physician and group leader at the University Hospital Zurich and the Center for Translational and Experimental Cardiology (CTEC). A cardiologist and translational researcher, his work focuses on the role of non-coding RNAs in HF, with particular emphasis on deep phenotyping. He received his clinical training in Germany and Switzerland at leading academic institutions. Dr. Kleeberger is passionate about mentoring the next generation of physicians and is committed to reducing the global burden of cardiovascular disease through integrated research and clinical innovation.

## Supplementary Material

qyaf166_Supplementary_Data

## Data Availability

No new data were generated or analysed in this study.
