# Supplementary material for: Seeing and sensing the heart: integrating non-coding RNA biomarkers with imaging in cardiovascular medicine
Source: Eur Heart J Imaging Methods Pract. 2026 Jan 9;4(1):qyaf166. doi: 10.1093/ehjimp/qyaf166 (PMC12825606; doi:10.1093/ehjimp/qyaf166)
Supplement: qyaf166_Supplementary_Data [file qyaf166_supplementary_data.docx]

**Supplementary references**

S51. Eyyupkoca F, Ercan K, Kiziltunc E, Ugurlu IB, Kocak A, Eyerci N. Determination of microRNAs associated with adverse left ventricular remodeling after myocardial infarction. Mol Cell Biochem. 2022;477(3):781-91.

S52. Gao W, Li CG, Yuan J, Zhang YM, Liu GB, Zhang JH, et al. Circ-MBOAT Regulates Angiogenesis via the miR-495/NOTCH1 Axis and Associates with Myocardial Perfusion in Patients with Coronary Chronic Total Occlusion. Int J Mol Sci. 2024;25(2):15.

S53. Limkakeng AT, Rowlette LL, Hatch A, Nixon AB, Ilkayeva O, Corcoran D, et al. A precision medicine approach to stress testing using metabolomics and microribonucleic acids. Pers Med. 2022:11.

S54. Hortmann M, Walter JE, Benning L, Follo M, Mayr RM, Honegger U, et al. Droplet digital PCR of serum miR-499, miR-21 and miR-208a for the detection of functionally relevant coronary artery disease. Int J Cardiol. 2019;275:129-35.

S55. Liu C-J, Chen J-j, Wu J-H, Chung Y-T, Chen J-W, Liu M-T, et al. Association of exosomes in patients with compromised myocardial perfusion on functional imaging. J Formos Med Assoc. 2024;123(9):968-74.

S56. Divakaran S, Randhawa V, Tahir UA, Robertson M, Waheed AA, Perillo A, et al. FDG PET/CT imaging and circulating biomarkers of inflammation in desmoplakin cardiomyopathy. ESC Heart Failure. 2025;12(2):1485-9.

S57. Nurmohamed NS, Cole JH, Budoff MJ, Karlsberg RP, Gupta H, Sullenberger LE, et al. Impact of atherosclerosis imaging-quantitative computed tomography on diagnostic certainty, downstream testing, coronary revascularization, and medical therapy: the CERTAIN study. Eur Heart J Cardiovasc Imaging. 2024;25(6):857-66.

S58. Koo BK, Yang S, Jung JW, Zhang J, Lee K, Hwang D, et al. Artificial Intelligence-Enabled Quantitative Coronary Plaque and Hemodynamic Analysis for Predicting Acute Coronary Syndrome. JACC Cardiovasc Imaging. 2024;17(9):1062-76.

S59. Luna Buitrago D, Jover E, Mameli E, et al. The loss of microRNA-26b promotes aortic calcification through the regulation of cell-specific target genes. Cardiovascular Research 2025;121:1778-1792.

S60. Liu W, Ling S, Sun W, et al. Circulating microRNAs correlated with the level of coronary artery calcification in symptomatic patients. Scientific Reports 2015;5:16099.

S61. Liu M, Wang L, Liu Z, et al. MiR-222-3p loaded stem cell nanovesicles repair myocardial ischemia damage via inhibiting mitochondrial oxidative stress. Life Sciences 2025;365:123447.

S62. Hou Q, Liu Y, Hou J, Song H, Zhang S, Zhang Y, et al. miR-3154: Novel Pathogenic and Therapeutic Target in Abdominal Aortic Aneurysm. Circulation Research. 2025;137(5):587-604.

S63. Desai, V. G., Kwekel JC, Vijay V, Moland CL, Herman EH, et al. (2014). Early biomarkers of doxorubicin-induced heart injury in a mouse model. Toxicology and Applied Pharmacology 281(2): 221-229.

S64. Roncarati R, Anselmi CV, Losi MA, Papa L, Cavarretta E, Martins PDC, et al. Circulating miR-29a, Among Other Up-Regulated MicroRNAs, Is the Only Biomarker for Both Hypertrophy and Fibrosis in Patients With Hypertrophic Cardiomyopathy. JACC. 2014;63(9):920-7.
